# Total Intermittent Pringle Maneuver during Liver Resection Can Induce Intestinal Epithelial Cell Damage and Endotoxemia

**DOI:** 10.1371/journal.pone.0030539

**Published:** 2012-01-24

**Authors:** Simon A. W. G. Dello, Kostan W. Reisinger, Ronald M. van Dam, Marc H. A. Bemelmans, Toin H. van Kuppevelt, Maartje A. J. van den Broek, Steven W. M. Olde Damink, Martijn Poeze, Wim A. Buurman, Cornelis H. C. Dejong

**Affiliations:** 1 Department of Surgery, Maastricht University Medical Center & Nutrim School for Nutrition, Toxicology and Metabolism, Maastricht University, Maastricht, the Netherlands; 2 Department of Biochemistry, Radboud University Nijmegen Medical Center, Nijmegen, the Netherlands; 3 Department of Surgery, Royal Free Hospital, and University College London, Division of Surgery and Interventional Science, London, United Kingdom; Duke University Medical Center, United States of America

## Abstract

**Objectives:**

The intermittent Pringle maneuver (IPM) is frequently applied to minimize blood loss during liver transection. Clamping the hepatoduodenal ligament blocks the hepatic inflow, which leads to a non circulating (hepato)splanchnic outflow. Also, IPM blocks the mesenteric venous drainage (as well as the splenic drainage) with raising pressure in the microvascular network of the intestinal structures. It is unknown whether the IPM is harmful to the gut. The aim was to investigate intestinal epithelial cell damage reflected by circulating intestinal fatty acid binding protein levels (I-FABP) in patients undergoing liver resection with IPM.

**Methods:**

Patients who underwent liver surgery received total IPM (total-IPM) or selective IPM (sel-IPM). A selective IPM was performed by selectively clamping the right portal pedicle. Patients without IPM served as controls (no-IPM). Arterial blood samples were taken immediately after incision, ischemia and reperfusion of the liver, transection, 8 hours after start of surgery and on the first post-operative day.

**Results:**

24 patients (13 males) were included. 7 patients received cycles of 15 minutes and 5 patients received cycles of 30 minutes of hepatic inflow occlusion. 6 patients received cycles of 15 minutes selective hepatic occlusion and 6 patients underwent surgery without inflow occlusion. Application of total-IPM resulted in a significant increase in I-FABP 8 hours after start of surgery compared to baseline (*p*<0.005). In the no-IPM group and sel-IPM group no significant increase in I-FABP at any time point compared to baseline was observed.

**Conclusion:**

Total-IPM in patients undergoing liver resection is associated with a substantial increase in arterial I-FABP, pointing to intestinal epithelial injury during liver surgery.

**Trial Registration:**

ClinicalTrials.gov NCT01099475

## Introduction

Intra-operative blood loss and red blood cell transfusions are associated with short- and long-term complications in liver surgery, such as operative mortality or major complications that require post-operative radiologic or surgical intervention [1], [2]. Intra-operative blood loss also predisposes patients to post-resectional liver failure [2]. In an attempt to avoid blood loss, the intermittent Pringle maneuver (IPM) is frequently applied in patients undergoing liver surgery. This implies intermittent clamping of the hepatoduodenal ligament, thereby occluding hepatic inflow [3].

IPM may have a negative effect on outcome after liver resection as a consequence of liver injury due to ischemia reperfusion (I/R) damage [4]. On the other hand, a short period of clamping of the portal triad is also used as a pre-conditioning method in order to protect the liver against ischemia damage when continuous clamping is performed [5], [6]. I/R damage of the liver as a consequence of IPM has been well studied, however little is known about the effects of IPM on the gut. Clamping the hepatoduodenal ligament causes stasis in the portal vein and the superior and inferior mesenteric veins, thereby reducing splanchnic outflow [7]. Intestinal hypoperfusion leads to enterocyte damage and gut barrier loss [8]. The effect of splanchnic hypoperfusion on intestinal damage as a consequence of IPM has been proven in several animal studies [9]–[11]. Sheen-Chen *et al.* [12] showed recently in rats that occlusion of the hepatoduodenal ligament significantly increased jejunal apoptosis. However, data on the effect of IPM on the gut in man are scarce. Loss of intestinal epithelial integrity is clinically important, as it is associated with the development of sepsis and multiple organ failure (MOF) following major surgery, trauma and shock [13], [14]. This might be especially important in patients with small for size liver remnant volume and/or parenchymal dysfunction due to neo-adjuvant chemotherapy, cholestasis and/or cirrhosis [15]–[17].

Intestinal fatty acid binding protein (I-FABP) is a small, water-soluble cytosolic protein that is easily released into the circulation upon enterocyte membrane integrity loss. I-FABP is solely present in mature epithelial cells of the small intestine and to a lesser extent in the large intestine [18]. We [8], [19] and others [18], [20], [21] have shown that I-FABP is an accurate marker for intestinal epithelial cell damage.

The principal aim of this study was to investigate whether IPM causes intestinal epithelial cell damage and barrier loss in patients undergoing liver resection.

## Methods

### Patients

Patients who underwent liver surgery at Maastricht University Medical Center were eligible for inclusion in this prospective trial. The study was approved by the medical ethical committee of Maastricht University Medical Center and conducted according to the revised version of the Declaration of Helsinki (October 2008, Seoul). All patients gave written informed consent.

Pre-operatively it was decided whether a Pringle maneuver was required according to the surgeon's preference. If a Pringle maneuver was required, patients were randomly assigned to IPM with 15 (15-IPM) or 30 minutes (30-IPM) ischemic intervals. Patients who did not require IPM served as controls (no-IPM). These patients were investigated in an RCT on a different topic by our group in the recent past [22]. The current study uses samples obtained from this previous RCT titled ‘Randomized controlled trial analyzing the effect of 15 or 30 min intermittent Pringle maneuver on hepatocellular damage during liver surgery’. The trial is registered at ClinicalTrials.gov - registration number: NCT01099475. No new randomization was carried out for this study. A subgroup of 6 selective intermittent Pringle maneuver patients (15 minutes ischemic intervals, sel-IPM) was added to the present study. The addition of these patients was approved by the above-mentioned IRB, and informed consent was obtained from each. The 15-IPM and 30-IPM groups were later pooled in a subanalysis to compare total-IPM and no-IPM.

### Operative procedure

Pre-operatively, all patients had radial artery and central venous catheters inserted to monitor arterial and central venous pressure as part of standard anesthetic care. Liver resection was performed as detailed elsewhere [23]. Resections were classified as major (≥3 segments) or minor (<3 segments or non-anatomical) resections. Laparotomy was performed by bilateral subcostal incision, followed by intraoperative ultrasonographic assessment of the liver. Once resectability had been confirmed, mobilization of the liver was performed to prepare for hepatic parenchymal transection, which was undertaken using a Cavitron Ultrasonic Surgical Aspirator (Force GSU System; Valleylab, Boulder, CO). Argon beam coagulation (Erbe, Germany, Tübingen), clips and sutures were used for hemostasis. Central venous pressure was maintained below 5 cm H_2_O during transection to reduce venous back-bleeding. The Pringle maneuver was performed by tightening a rubber tube around the entire hepatoduodenal ligament (total Pringle maneuver) or selectively around the left or right portal pedicle using an extra Glissonian approach [24]. Cycles of 15 or 30 minutes of occlusion were alternated with 5 minutes of reperfusion. The control and Pringle manoeuvre patients were operated by the same surgeons.

### Blood sampling and processing

**Figure 1:**
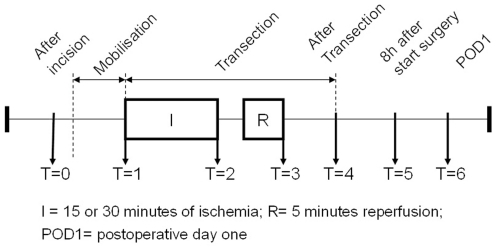
Timeline of sample collection.

Arterial blood samples were obtained from the radial artery line at predefined time points (figure 1). Blood samples were collected in pre-chilled EDTA containing vacuum tubes (BD vacutainer, Becton Dickinson Diagnostics, Aalst, Belgium) and kept on ice. Blood was centrifuged in a pre-chilled centrifuge at 4°C (3500 rotations per minute, 15 minutes). Plasma was immediately stored at −80°C until batch analysis. All analyses were performed by one person after completion of patient inclusion. Patients were admitted to the hospital one day pre-operatively and routine blood tests were performed by the clinical chemistry department.

### I-FABP measurement

I-FABP is a highly sensitive and specific marker of intestinal epithelial cell damage [19]. I-FABP plasma levels were determined using an in-house enzyme-linked immunosorbent assay (ELISA) that selectively detects human I-FABP (range: 10–1280 pg/ml). Due to its small molecular weight, plasma I-FABP passes the glomerular filter (fractional renal excretion: 28%, half-life time: 11 minutes) [25].

### Source and fate of I-FABP

In order to measure the source and fate of I-FABP, blood was also drawn from the portal vein and hepatic vein after liver transection in the no-IPM and total-IPM group (T = 4, figure 1). Arterio-venous (AV) differences of I-FABP across the gut and the hepatosplanchnic area (gut+liver) were calculated. AV-differences of I-FABP were calculated using the following formulae:

AV-difference gut = portal venous [I-FABP]−arterial [I-FABP]

Hepatosplanchnic AV-difference = hepatovenous [I-FABP]−arterial [I-FABP].

### EndoCAb measurement

IgG Endotoxin Core Antibodies were used to quantify endotoxemia. A drop from pre-operative values to post-operative values was interpreted as consumption of antibodies to endotoxin by systemic release of endotoxin. IgG EndoCAb was measured using a commercially available enzyme-linked immunosorbent assay (ELISA), kindly provided by Hycult Biotechnology, Uden, the Netherlands (range: 0.13–8.00 GMU/ml). EndoCAb data are expressed in General Median Units (GMU)/ml. General Median Units of IgG are arbitrary and are based on medians of healthy adults, with 100 GMU/ml being the median.

### Statistics

Mann Whitney *U* test was applied for two group comparison for continuous data. Wilcoxon signed rank test was applied for pairwise comparison for continuous data. Dichotomous data were compared using Fisher exact test. Multiple group comparisons for continuous data were done by Kruskal-Wallis test, with Dunn's post hoc test. All data are expressed as median and range. A *p*-value<0.05 was considered statistically significant. Statistical analysis was performed using Prism 5.0 for Windows (Graphpad software, Inc, San Diego, CA).

## Results

### Patients

Twenty-four patients (11 females; 13 males) scheduled for hepatectomy for primary (n = 2) or secondary malignant liver tumours (n = 22) were included. Thirteen patients underwent major liver resections (≥3 segments) and eleven a minor liver resection (<3 segments). The 15-IPM group received 15 minutes of ischemia (n = 7), median 2 (2–5) cycles and a cumulative total ischemia time of 33 (30–75) minutes. The 30-IPM group received 30 minutes of ischemia (n = 5), median 1 (1–2) cycle and a cumulative total ischemia time of 30 (30–56) minutes.

The sel-IPM group received selective clamping of the right portal pedicle (n = 6), and controls (no-IPM) received no vascular clamping (n = 6). There were no relevant significant differences between groups neither in baseline characteristics (table 1) nor in operation time, intra-operative blood loss, extent of resection and post-operative creatinin levels (table 2). There were no significant differences in pre- and post-operative creatinin levels (table 1,2).

**Table 1:** Patient characteristics.

	15 min total-IPM(n = 7)	30 min total-IPM(n = 5)	No-IPM(n = 6)	Sel-IPM(n = 6)	*p*
Age (years)	60.8 (48.3–79.9)	67.3 (60.3–77.4)	60.5 (59.6–70.1)	64.6 (42.9–70.1)	0.64
Gender	(3 F; 4 M)	(2 F; 3 M)	(1 F; 5 M)	(5 F; 1 M)	0.14
Height (cm)	1.77 (1.55–1.92)	1.70 (1.63–1.75)	1.76 (1.72–1.95)	1.65 (1.60–1.86)	0.11
Weight (kg)	72 (56–100)	74 (54–83)	75 (68–90)	71 (55–88)	0.83
Body Mass Index	23.2 (22.6–27.4)	24.2 (20.3–28.7)	23.4 (23.0–25.7)	25.1 (19.3–30.8)	0.70
Aspartate-aminotransferase (IU/L)	33 (13–52)	16 (7–25)	21 (10–32)	19 (11–26)	0.13
Alanine-aminotransferase (IU/L)	36 (11–51)	26 (8–41)	26 (7–55)	23 (21–29)	0.75
Lactate dehydrogenase (IU/L)	399 (305–595)	356 (299–432)	319 (291–557)	389 (316–514)	0.55
Gamma-glutamyl transpeptidase (IU/L)	56 (22–204)	37 (32–169)	34 (18–83)	33 (29–63)	0.61
Alkaline phosphatase (IU/L)	134 (55–256)	90 (66–124)	80 (58–128)	115 (57–126)	0.52
Bilirubin (µM)	13.8 (11.3–14.2)	10.6 (8.3–13.0)	14.0 (6.9–16.5)	11.3 (7.8–12.9)	0.15
Pre-operative creatinin (µmol/L)	78 (59–125)	92 (85–137)	76 (54–96)	80.5 (46–287)	0.40

Data are presented as median (range). All data are preoperative values.

**Table 2:** Characteristics of surgical procedures.

	15 min total-IPM(n = 7)	30 min total-IPM(n = 5)	No-IPM(n = 6)	Sel-IPM(n = 6)	*p*
Operation time(hours: minutes)	3:15 h (2:10–6:30)	4:15 h (3:09–4:45)	3:24 h (2:20–4:10)	3:45 h (2:27–4:30)	0.62
Blood loss (ml)	850 (250–3900)	1000 (250–2500)	750 (200–2600)	1050 (400–2500)	0.93
Number of resected segments	3 (2–3)	3(1–3)	2 (1–3)	3 (2–3)	0.48
Post-operative creatinin (µmol/L) day 0/1	71 (55–114)	110 (101–115)	88 (52–93)	76 (44–248)	0.17

Data are presented as median (range).

### Plasma baseline I-FABP values

Baseline (T = 1) arterial I-FABP levels did not differ significantly between groups (15-IPM, 532 pg/ml [353–1,078]; 30-IPM, 565 pg/ml [500–1,156]; sel-IPM, 874 pg/ml [478–1,198]; no-IPM, 502 pg/ml [161–966] *p = 0.26*).

### Intestinal epithelial injury in 15 min vs. 30 min total IPM

No significant differences between 15-IPM and 30-IPM groups in median plasma I-FABP values were found at any time point. In the 15 minutes IPM group a significant increase in I-FABP was observed from baseline (T = 1) to after transection (T = 4) (15-IPM: 532 pg/ml [353–1,078] to 891 pg/ml [392–3,053] *p*<0.05) and from baseline (T = 1) to 8 hours after start of surgery (T = 5) (15-IPM: 532 pg/ml [353–1,078] to 1,478 pg/ml [627–2,000] *p*<0.05). Application of 30 minutes cycles of inflow occlusion did not significantly increase the release of I-FABP (30-IPM: 597 pg/ml [500–1,156] to 1,077 pg/ml [560–1,664], *p* = 0.19).

### Intestinal epithelial injury in total IPM vs. control

**Figure 2:**
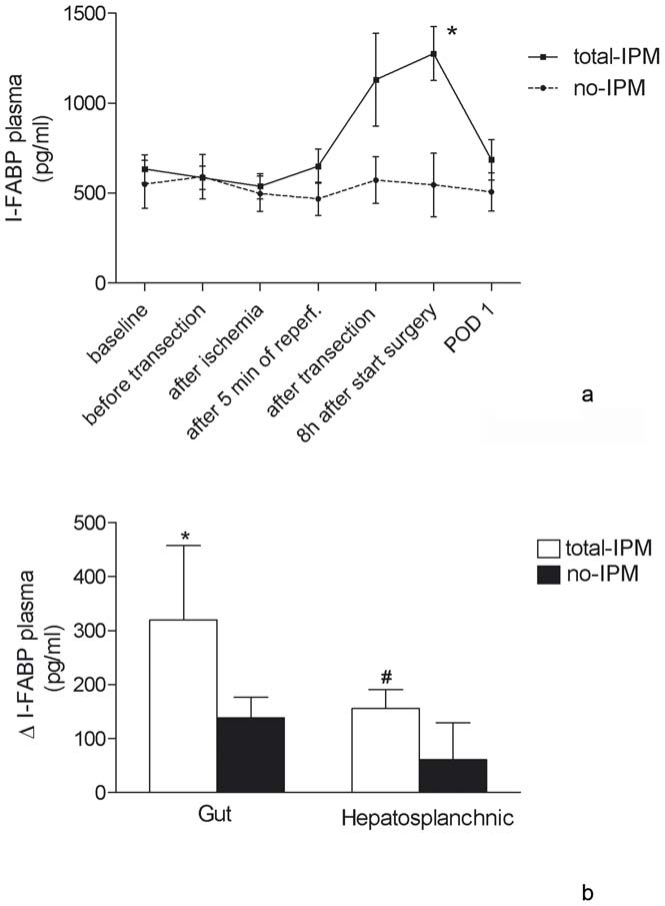
Time course of I-FABP plasma levels and interorgan arterio-venous concentration differences. **2A** For visual purposes data were plotted as mean and SEM. * *p*<0.005 compared to baseline of total-IPM (T = 0), *p*<0.01 compared to no-IPM on T = 5. **2B** Mean (SEM) arterio-venous concentration gradients of I-FABP across the gut (portal venous minus arterial) and the hepatosplanchnic area (hepatic venous minus arterial). I-FABP was specifically released from the gut (*p<0.0001 vs. zero) and this resulted in a net I-FABP release from the hepatosplanchnic area (^#^ *p*<0.005 vs. zero).

The 15-IPM and 30-IPM groups were subsequently pooled and compared with controls (no-IPM) since there was no significant difference in intestinal epithelial cell damage between 15-IPM and 30-IPM. In the total-IPM group, plasma I-FABP levels increased significantly from baseline to 8 hours after start of surgery (549 pg/ml [353–1,156] to 1,279 pg/ml [560–2,000], *p*<0.005). In the no-IPM group, no significant differences were observed in I-FABP concentrations between the different time points. Consequently, plasma I-FABP levels 8 hours after start of surgery were significantly higher in the total-IPM group compared to the no-IPM group (respectively, 1,279 pg/ml [560–2,000] and 413 pg/ml [245–1,388], *p*<0.01) (figure 2A).

### Intestinal epithelial injury in sel-IPM

In patients who received selective clamping of the right portal pedicle (sel-IPM), plasma I-FABP levels did not increase significantly from baseline to 8 hours after start of surgery (806 pg/ml [478–1,198] to 924 pg/ml [248–2,823], *p* = 0.31). Moreover, there were no significant differences at any time point between sel-IPM and no-IPM.

### Organ specific I-FABP release

In order to prove that I-FABP is specifically released from the gut we performed an organ balance analysis to reveal the origin of circulating I-FABP. The data show that I-FABP was specifically released from the gut after liver transection. This resulted in a net release of I-FABP from the hepatosplanchnic area (figure 2B).

### Determination of endotoxemia

**Figure 3:**
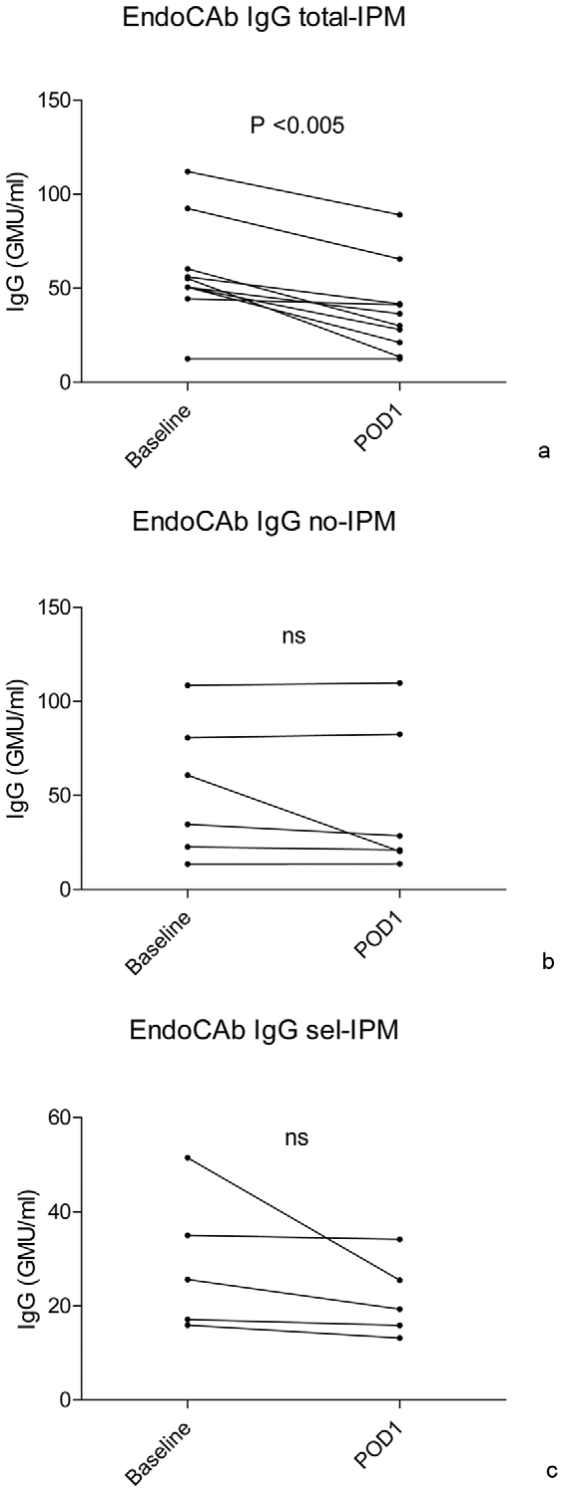
Values are median (range), pairwise comparisons of EndoCAb at baseline (T = 0) and on post-operative day one (T = 6). **3A** Total-IPM: two patients were excluded from this analysis because of missing samples on post-operative day 1. EndoCAb levels of one patient were undetectable and therefore the lower detection limit of the EndoCAb ELISA kit, corrected for dilution factor was used (12.5 GMU/ml). **3B** No-IPM. **3C** Sel-IPM: 1 patient was excluded from analysis because of missing samples on post-operative day 1.

Plasma levels of natural IgG against endotoxin were significantly decreased in the total-IPM group on post-operative day 1 (POD1) compared to baseline (baseline, 52.9 GMU/mL [10.0–112.1], POD1, 33.2 GMU/mL [10.0–89.1] *p*<0.005). In the no-IPM and sel-IPM group, no significant decrease from baseline to POD1 was observed (figure 3).

## Discussion

This study aimed to investigate whether IPM causes loss of intestinal epithelial cell integrity and leads to endotoxemia in patients undergoing liver resection. The results of the present study show that the use of total IPM is associated with intestinal epithelial cell damage and subsequent endotoxemia. In the total-IPM group (15 min and 30 min total IPM combined), plasma I-FABP levels were significantly increased 8 hours after start of surgery compared to baseline, while in the no-IPM group and sel-IPM group no significant differences in I-FABP levels were observed between different time points. By measuring concentration differences across the gut and the hepatosplanchnic area, we were able to show that there was a net I-FABP release from the hepatosplanchnic area in the total-IPM group, explaining the high levels of I-FABP in this group.

In the total-IPM group IgG EndoCAb decreased significantly on POD1 compared to baseline while this effect was not observed in the no-IPM and sel-IPM group. Consumption of IgG EndoCAb in the total IPM group suggests that total IPM resulted in translocation of gut derived endotoxins, possibly by intestinal barrier dysfunction due to epithelial cell damage.

The results of the present study are in line with several animal studies. Ochiai *et al.* [9] showed that IPM caused intestinal epithelial cell damage and increased small intestinal permeability in rats. In two other rat studies, increased bacterial translocation [10] and endotoxemia [10], [11] were demonstrated after IPM. Data on the effect of IPM on the gut in man are scarce. It remains unknown whether it is only the impaired microcirculation in the intestine during portal clamping which causes cell damage or that the temporal acute rise of venous pressure in the mesenteric system in itself also plays a role. King *et al.* [26] showed that when splanchnic venous outflow was occluded in patients, both intestinal oxygen extraction ratio and portal venous lactate increased. An acute rise of venous pressure in the microcirculation in itself will probably also impair perfusion and consequently cause tissue hypoxia. It is most likely a combination of the two mechanisms that explains the high levels of I-FABP in these patients. It would be worthwhile to study the microcirculation in vivo during the total IPM in order to further explore the pathophysiological mechanism behind the intestinal cell damage in these patients. In this context Ferri *et al.* [27] showed that bacterial translocation to mesenteric lymph nodes occurred during liver resection in patients under continuous inflow occlusion, but there was no correlation between positive lymph nodes and post-operative infectious complications.

Patients undergoing liver resection are more susceptible to development of a systemic inflammatory response due to endotoxemia, as hepatic endotoxin clearance is compromised due to a reduction of Kuppfer cells [28]. This is caused by reduction of the functional hepatic liver mass and by ischemia reperfusion damage to the functional liver parenchyma when IPM is applied. Translocation of intraluminal intestinal toxins as a consequence of intestinal epithelial cell damage due to total-IPM could further contribute to systemic endotoxemia. It may therefore play an important role in the pathogenesis of the systemic inflammatory response syndrome (SIRS) and sepsis in patients undergoing liver resection. In line with this, infective complications are reported to negatively affect both short- and long-term outcome after liver resection [29], [30]. This might be especially important in patients with underlying liver disease and cholestasis. In the latter, most often infected areas of non-optimally drained liver segments are present during surgery [31]. To prove these assumptions it would be worthwhile to investigate in future studies whether there is a relation between infective complications and the application of the total-IPM.

The present study sheds new light on the question whether performing IPM is favorable in patients undergoing liver surgery. Hepatectomies without IPM can be performed safely due to advances in liver surgery such as the development of modern hemostatic devices and improvements in anesthesiological management [32]. Acute major bleeding remains an indication for IPM, but current evidence shows no benefit for IPM on outcome after liver resection [33]. Therefore in the modern era of liver surgery systematic use of IPM has become more often a subject of debate. Our data show that total-IPM is associated with intestinal epithelial cell damage and endotoxemia. This could play a role in the pathophysiology of infective postoperative complications. It is clinically relevant that the role of gut integrity loss induced by ischemia/reperfusion of the liver during liver resections and liver transplantations is further investigated in future studies. This pathophysiologic mechanism is probably underestimated in these patients. Unravelling this mechanism could help to (preoperatively) identify patients with a higher risk of infective postoperative complications.

A possibly more safe approach than total IPM is selective IPM, which is performed by selectively excluding the right or left hemi liver from the circulation. Selectively clamping the right or left portal pedicle is safe and feasible for patients with normal liver parenchyma and especially in cirrhotic patients the selective IPM induces less ischemic liver injury compared to total IPM [34]. One would expect that also the intestinal epithelial cell integrity is less compromised in these patients because splanchnic outflow is only partly reduced. This hypothesis was confirmed in the present study as no significant increase was observed in arterial I-FABP levels in 6 patients undergoing selective IPM. However, there were no significant differences on any time point between the sel-IPM group and the total-IPM group. This is probably due to the relatively small group size (type II error), as only one patient showed a substantial increase in I-FABP.

The present study shows that the use of the total intermittent Pringle maneuver causes intestinal epithelial cell damage and endotoxemia during liver surgery in man. IPM can therefore potentially increase the risks of liver resections despite a possible reduction of intraoperative blood loss. Intestinal epithelial cell damage and endotoxemia induced by IPM could negatively affect the patient's condition and post-operative recovery. Whether gut damage as a consequence of the total-IPM is causally related to systemic inflammation remains to be established.

## References

[1] Imamura H, Seyama Y, Kokudo N, Maema A, Sugawara Y (2003). One thousand fifty-six hepatectomies without mortality in 8 years.. Arch Surg.

[2] Kooby DA, Stockman J, Ben-Porat L, Gonen M, Jarnagin WR (2003). Influence of transfusions on perioperative and long-term outcome in patients following hepatic resection for colorectal metastases.. Ann Surg.

[3] Pringle JH (1908). V. Notes on the Arrest of Hepatic Hemorrhage Due to Trauma.. Ann Surg.

[4] Patel A, van de Poll MC, Greve JW, Buurman WA, Fearon KC (2004). Early stress protein gene expression in a human model of ischemic preconditioning.. Transplantation.

[5] Clavien PA, Selzner M, Rudiger HA, Graf R, Kadry Z (2003). A prospective randomized study in 100 consecutive patients undergoing major liver resection with versus without ischemic preconditioning.. Ann Surg.

[6] Clavien PA, Yadav S, Sindram D, Bentley RC (2000). Protective effects of ischemic preconditioning for liver resection performed under inflow occlusion in humans.. Ann Surg.

[7] Belghiti J, Marty J, Farges O (1998). Techniques, hemodynamic monitoring, and indications for vascular clamping during liver resections.. J Hepatobiliary Pancreat Surg.

[8] Derikx JP, van Waardenburg DA, Thuijls G, Willigers HM, Koenraads M (2008). New Insight in Loss of Gut Barrier during Major Non-Abdominal Surgery.. PLoS One.

[9] Ochiai H, Nakamura S, Suzuki S, Baba S (1997). Pancreatic damage resulting from temporary portal triad interruption during partial hepatectomy: protective effect of a prostaglandin I2 analogue.. J Surg Res.

[10] Filos KS, Kirkilesis I, Spiliopoulou I, Scopa CD, Nikolopoulou V (2004). Bacterial translocation, endotoxaemia and apoptosis following Pringle manoeuvre in rats.. Injury.

[11] Unno N, Uchiyama T, Yamamoto N, Inuzuka K, Sagara D (2006). Portal triad occlusion induces endotoxin tolerance: role of portal congestion.. J Surg Res.

[12] Sheen-Chen SM, Su FI, Tang RP, Huang CC, Eng HL (2011). Cellular changes in hepatocytes and intestinal endothelium after hepatoduodenal ligament occlusion and protective effects of caspase inhibition.. Ann Surg.

[13] Derikx JP, Poeze M, van Bijnen AA, Buurman WA, Heineman E (2007). Evidence for intestinal and liver epithelial cell injury in the early phase of sepsis.. Shock.

[14] Holland J, Carey M, Hughes N, Sweeney K, Byrne PJ (2005). Intraoperative splanchnic hypoperfusion, increased intestinal permeability, down-regulation of monocyte class II major histocompatibility complex expression, exaggerated acute phase response, and sepsis.. Am J Surg.

[15] Schindl MJ, Redhead DN, Fearon KC, Garden OJ, Wigmore SJ (2005). The value of residual liver volume as a predictor of hepatic dysfunction and infection after major liver resection.. Gut.

[16] Vauthey JN, Pawlik TM, Ribero D, Wu TT, Zorzi D (2006). Chemotherapy regimen predicts steatohepatitis and an increase in 90-day mortality after surgery for hepatic colorectal metastases.. J Clin Oncol.

[17] Poon RT, Fan ST (2004). Hepatectomy for hepatocellular carcinoma: patient selection and postoperative outcome.. Liver Transpl.

[18] Pelsers MM, Namiot Z, Kisielewski W, Namiot A, Januszkiewicz M (2003). Intestinal-type and liver-type fatty acid-binding protein in the intestine. Tissue distribution and clinical utility.. Clin Biochem.

[19] Derikx JP, Vreugdenhil AC, Van den Neucker AM, Grootjans J, van Bijnen AA (2009). A pilot study on the noninvasive evaluation of intestinal damage in celiac disease using I-FABP and L-FABP.. J Clin Gastroenterol.

[20] Kanda T, Fujii H, Tani T, Murakami H, Suda T (1996). Intestinal fatty acid-binding protein is a useful diagnostic marker for mesenteric infarction in humans.. Gastroenterology.

[21] Kanda T, Tsukahara A, Ueki K, Sakai Y, Tani T (2011). Diagnosis of ischemic small bowel disease by measurement of serum intestinal fatty acid-binding protein in patients with acute abdomen: a multicenter, observer-blinded validation study.. J Gastroenterol.

[22] van den Broek MA, Bloemen JG, Dello SA, van de Poll MC, Olde Damink SW (2011). Randomized controlled trial analyzing the effect of 15 or 30 minutes intermittent Pringle manoeuvre on hepatocellular damage during liver surgery.. J Hepatol.

[23] Dejong C, Garden O, Majid AA, Kingsnorth A (2003). Neoplasms of the liver.. Advanced surgical practice.

[24] Topal B, Aerts R, Penninckx F (2007). Laparoscopic intrahepatic Glissonian approach for right hepatectomy is safe, simple, and reproducible.. Surg Endosc.

[25] van de Poll MC, Derikx JP, Buurman WA, Peters WH, Roelofs HM (2007). Liver manipulation causes hepatocyte injury and precedes systemic inflammation in patients undergoing liver resection.. World J Surg.

[26] King KL, Tang GJ, Wu CW, Lui WY (2001). Ischaemic change of the human intestine after total portal occlusion during liver resection.. S Afr J Surg.

[27] Ferri M, Gabriel S, Gavelli A, Franconeri P, Huguet C (1997). Bacterial translocation during portal clamping for liver resection. A clinical study.. Arch Surg.

[28] Gross K, Katz S, Dunn SP, Cikrit D, Rosenthal R (1985). Bacterial clearance in the intact and regenerating liver.. J Pediatr Surg.

[29] Cescon M, Vetrone G, Grazi GL, Ramacciato G, Ercolani G (2009). Trends in perioperative outcome after hepatic resection: analysis of 1500 consecutive unselected cases over 20 years.. Ann Surg.

[30] Farid SG, Aldouri A, Morris-Stiff G, Khan AZ, Toogood GJ (2010). Correlation between postoperative infective complications and long-term outcomes after hepatic resection for colorectal liver metastasis.. Ann Surg.

[31] Cherqui D, Benoist S, Malassagne B, Humeres R, Rodriguez V (2000). Major liver resection for carcinoma in jaundiced patients without preoperative biliary drainage.. Arch Surg.

[32] Chouillard EK, Gumbs AA, Cherqui D (2010). Vascular clamping in liver surgery: physiology, indications and techniques.. Ann Surg Innov Res.

[33] Rahbari NN, Wente MN, Schemmer P, Diener MK, Hoffmann K (2008). Systematic review and meta-analysis of the effect of portal triad clamping on outcome after hepatic resection.. Br J Surg.

[34] Figueras J, Llado L, Ruiz D, Ramos E, Busquets J (2005). Complete versus selective portal triad clamping for minor liver resections: a prospective randomized trial.. Ann Surg.

